# Bioavailability and Bioefficacy of Hemp By-Products in Ruminant Meat Production and Preservation: A Review

**DOI:** 10.3389/fvets.2020.572906

**Published:** 2020-09-25

**Authors:** Farouk Semwogerere, Chenaimoyo L. F. Katiyatiya, Obert C. Chikwanha, Munyaradzi C. Marufu, Cletos Mapiye

**Affiliations:** ^1^Department of Animal Sciences, Faculty of AgriSciences, Stellenbosch University, Stellenbosch, South Africa; ^2^Department of Veterinary Tropical Diseases, Faculty of Veterinary Science, University of Pretoria, Pretoria, South Africa

**Keywords:** bioactive profile, digestibility, hempseed, meat quality, shelf life

## Abstract

Plant by-products obtained from agro-industrial processes require valorisation to demonstrate their potential for enhancing animal health, meat production, and shelf life extension. One example is the fast-growing hemp industry, which produces seeds, leaves, seed oil, and cake. Studies on the nutritional value of hempseed cake have shown it can be a valuable source of protein in ruminant diets. However, there is limited documentation on the bioavailability and bioefficacy of hemp phytochemicals for improving ruminant health, production, and extending meat shelf life. The current review provides an overview of existing information on nutrient and phytochemical composition of hemp by-products, their bioavailability, and bioefficacy, and explores current limitations and prospects regarding their valorisation.

## Introduction

**Figure 1:**
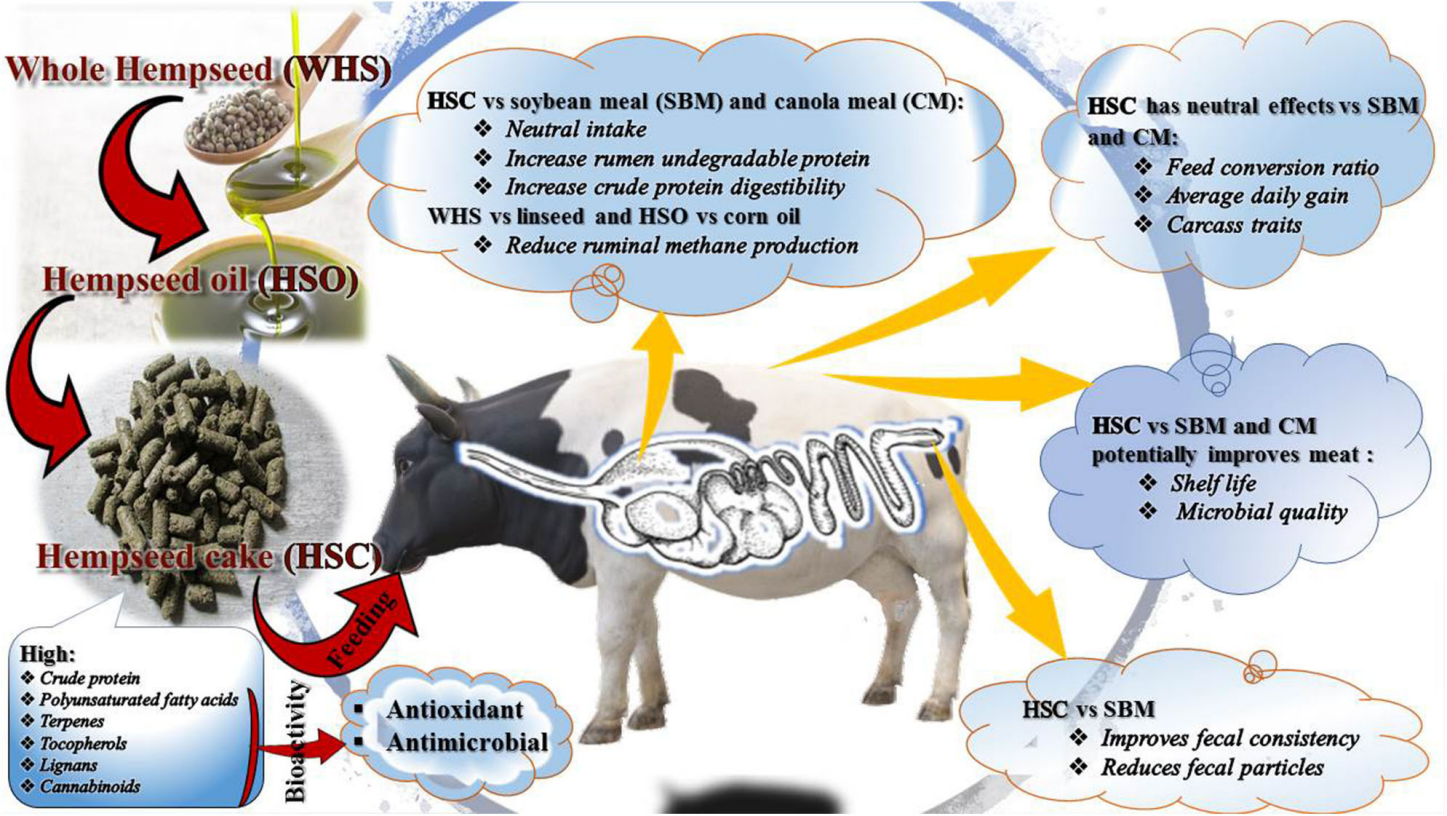
Valorisation of hemp by-products nutrients and bioactive compounds in ruminants.

Research into novel and underutilized feed resources for ruminant production and shelf life enhancement is paramount to sustainability of livestock and meat industries (1). Among the novel alternatives to conventional feed resources are hemp (*Cannabis sativa* L.) by-products (i.e., seed, oil, oilseed cake, hulls, and leaves) (2, 3). Growing legalization and demand are anticipated to increase global production of hemp and its by-products (4, 5). As a consequence, the feed and meat industries could benefit provided hemp by-products can be valorised as feed ingredients and biopreservatives (Figure 1).

There are few reports on incorporation of hemp by-products into ruminant diets (6, 7). In Europe, inclusion of hempseed cake (HSC) has been restricted to <50 g/kg DM in ruminant diets (2). In other jurisdictions such as North America, feeding of hemp by-products awaits approval and will be done based on applications for individual by-products (8, 9). This is partly because of limited data on the bioavailability of dominant bioactive compounds of Cannabis species (i.e., tetrahydrocannabinol, THC, and cannabidiol, CBD) in ruminant animals (2) and the known psychoactive effects of THC in humans (10). In addition, there is limited knowledge on the bio-efficacy of these and other bio-actives in a meat matrix (2). The present review explores the composition, bioavailability and bioefficacy of hemp by-product nutrients and bioactives in ruminant meat production and preservation. Challenges and opportunities for valorisation of hemp by-products for meat production and shelf life extension are also discussed.

## Global Hemp Production and Utilization

Globally, the FAO estimates of 32 square kilometers (km^2^) of hemp are harvested including 143 metric tons (MT) hempseed, 50 MT seed oil, 93 MT cake, and 331 MT leaves are produced mainly from France, China, and Chile (Table 1), but this does not include Canada or the USA, which are also major producers with an estimated 315 km^2^ (23) and 1,160 km^2^ (24) under cultivation, respectively. Overall, scant data exists on hemp or by-products production, especially in Africa. Hemp is a multifaceted plant commonly cultivated for fiber and oil, although other components of the plant might have beneficial uses as medicine (25, 26). Primary uses of hemp are determined by variety and region of origin (4). The majority of hemp varieties are cultivated for seed production, of which, hempseed oil is the primary valuable output (14, 15). On average, hempseed has 30–35% seed oil, that is extracted only by cold pressing (11), with HSC being the main solid by-product of oil extraction. Cold-press method preserves physical and chemical quality of oil (15). A small proportion (i.e., 0.4–10%) of the oil is retained in HSC after extraction (15). Hemp stems are utilized in the textile, livestock (i.e., beddings), and automotive industries as they utilize a large amount of fiber (11, 27). Hemp leaves and inflorescences are sources of bioactive compounds used in pharmaceuticals and human foods (11, 28).

**Table 1:** Global production of hemp and its by-products.

		**Production (tons)**			
**Country**	**Area harvested (ha)**	**Seed**	Oil^*^	Cake^*^	Leaf meal^**^
France	16,511	125,362	43,877	81,485	170,063
Russian	4,691	2,117	741	1,376	48,317
China	4,342	11,822	4,138	7,684	44,723
Chile	2,660	1,533	537	996	27,398
Hungary	1,606	390	137	254	16,542
Ukraine	1,133	596	209	387	11,670
Romania	799	84	29	55	8,230
Iran	193	198	69	129	1,988
Spain	140	750	263	488	1,442
Poland	59	28	10	18	608
Turkey	6	3	1.05	1.95	61.8

* *Oil was estimated at 35% seed content and resultant cake (11)*.

** *Leaf meal was estimated at 10.3 tons/ha (12)*.

*Source: (13)*.

Hemp varieties are rarely used for medicinal purposes because they have low THC content (<0.2%) (11). Hemp production is low as it is often confused with marijuana which is illegal to cultivate in most countries (14, 15). However, many countries have legalized the commercial production of hemp and utilization of its by-products (26). For example, South Africa recently passed a law to license cultivation and processing of hempseed using varieties with <0.001% THC (29). The liberalization of hemp legalization by many countries is likely to increase oil production, and consequent utilization of its by-products (i.e., HSC and leaf meals). This increase in oil, HSC, and leaf meal could potentially be beneficial to hemp processing, animal feed and meat industries. Currently, hemp by-products are not recognized as commercial livestock feed ingredients in most countries, even though some have come up with inclusion guidelines for livestock diets (2). Generally, limited literature exists on the utilization of hemp by-products except for the oil (11, 30). The value of hemp by-products as animal feed lies in the nutritional and phytochemical contents of HSC, especially when leaf production is limited.

## Nutrient Composition of Hemp By-Products

Chemical composition of hemp by-products is largely influenced by variety, pressing, and seed treatment methods (15, 31). However, hemp by-product chemical composition is generally similar to soybean meal (SBM) except for hurds, which contains extremely low CP and ether extract (EE; Table 2). The CP content of hemp by-products is greater than the endorsed dietary requirements for maintenance (60–110 g/kg CP DM) and growth (120–180 g/kg CP DM) of ruminants (32, 33, 36). Hemp by-products have a well-balanced amino acid profile comparable to SBM, with tryptophan as a limiting amino acid (Table 2). However, hemp by-products are deficient in growth-limiting amino acids including methionine (1.8 and 2.0% CP) and lysine (6.4 and 6.8% CP) as per the body requirements for goats and cattle, respectively (36, 37). The EE, neutral detergent fiber (NDF) and acid detergent fiber (ADF) of hempseed, cake, and hulls are greater than SBM (Table 2). The difference in EE content of hemp by-products and SBM could be attributed to the oil extraction method. Solvent extraction has greater oil extraction efficiency than cold press which leaves about 7% of the oil in the cake (38). The EE value of hempseed, cake and hulls is thus about 2.5 times greater than the EE (<50 g/kg DM) recommended for optimal ruminant production (39). This high EE content might affect the inclusion level of hemp by-products in ruminant diets.

**Table 2:** Chemical (g/kg DM, Mean ± SD) and amino acid (%, Mean ± SD) composition of hemp by-products.

**Chemical composition**	**Hemp by-product**					**Soybean meal**
	**Seed**	**Cake**	**Hulls**	**Hurds**	**Leaves**	
DM	928 ± 16.52	929 ± 16.1	949 ± 18	963	931	906 ± 9.9
CP	260 ± 48.64	341 ± 50.4	127 ± 37	32.0	238	503 ± 18.4
EE	290 ± 111.24	116 ± 15.5	103 ± 58	0.08	200	40 ± 15.9
NDF	328 ± 28.92	395 ± 40.7	649 ± 93	900	–	125 ± 17.6
ADF	230 ± 15.72	275 ± 19.3	502 ± 61	789	–	89 ± 10.2
Ash	57 ± 10.85	68 ± 3.44	39 ± 60	–	112	69 ± 5.5
**Amino acid**						
Arginine	2.42 ± 0.26	4.11 ± 0.69	0.94 ± 0.80	–	4.32	3.63 ± 0.21
Cystine	0.44 ± 0.06	0.74 ± 0.15	0.18 ± 0.06	–	0.79	0.71 ± 0.06
Histidine	0.58 ± 0.06	0.98 ± 0.19	0.25 ± 0.15	–	2.21	1.27 ± 0.08
Isoleucine	0.90 ± 0.11	1.52 ± 0.23	0.39 ± 0.14	–	3.23	2.47 ± 0.45
Leucine	1.58 ± 0.16	2.47 ± 0.23	0.71 ± 0.27	–	7.1	3.79 ± 0.18
Lysine	0.91 ± 0.09	1.39 ± 0.27	0.33 ± 0.16	–	3.84	3.11 ± 0.16
Methionine	0.60 ± 0.08	0.93 ± 0.25	0.18 ± 0.12	–	0.89	0.65 ± 0.06
Phenylalanine	1.09 ± 0.16	1.70 ± 0.30	0.53 ± 0.09	–	3.94	2.68 ± 0.46
Threonine	1.07 ± 0.22	1.42 ± 0.23	0.36 ± 0.13	–	2.26	1.96 ± 0.10
Tryptophan	0.24 ± 0.06	0.41 ± 0.10	0.06 ± 0.04	–	–	0.71 ± 0.06
Valine	1.21 ± 0.14	2.01 ± 0.30	0.60 ± 0.31	–	3.91	2.46 ± 0.30

*Sources: (2, 3, 6, 7, 14–22)*.

High inclusion levels of hemp by-products have been recommended for ruminants based on NDF content (2). More so, hemp by-products are within the recommended dietary NDF content of 150–300 g/kg DM required for optimal ruminant production (40, 41). Lignin content of whole hempseed and HSC has been reported to be 112–117 g/kg DM (16, 42). NRC (36) suggested that ruminant dietary lignin content above 40 g/kg DM will probably decrease DM intake and digestibility. High lignin content of HSC could be attributed to the hull (30–46% of seed) remains in the cake during oil extraction (4, 34, 38). More so, hulls contain the highest (65%) fiber portion of the hempseed (5, 15). In hempseed, lignin is only found in the hulls (4).

Metabolisable energy (ME) of HSC ranges from 9.21 to 13.01 MJ/kg DM (6, 17). These values exceed the average requirements for maintenance (i.e., 0.424, 0.401, and 0.497 MJ ME/kg BW^0.75^) and growth (0.03, 0.015, and 0.016 MJ ME/g of weight gain) for goats, sheep, and cattle, respectively (32, 36) when HSC fed at 2% BW, hence, HSC could serve well as a ruminant feedstuff. The mineral elements of hemp by-products are lower than ruminant maintenance requirements though the whole seed meets micro-mineral requirements (Table 3). Although their mineral content is not well-researched, all hemp by-products except hurds can be used as potential feed ingredients in ruminant diets.

**Table 3:** Hemp by-products mineral content and ruminant requirements (Mean ± SD).

	**Hempseed by-product**			**Nutrient requirements**	
**Minerals**	**Seed**	**Oil**	**Cake**	**Small ruminants**	**Cattle**
**Macro (g/kg DM)**					
Na	–	0.09	0.09	0.85 ± 0.12	1.00 ± 0.28
Ca	3.1 ± 2.3	0.05	1.91	4.20 ± 3.96	6.50 ± 6.36
P	–	–	28.0	1.95 ± 1.48	2.40 ± 1.98
Mg	3.6 ± 0.8	0.20	2.31	1.05 ± 0.21	1.75 ± 0.64
K	10.6 ± 6.6	0.02	5.06	5.00 ± 0.00	5.00 ± 0.00
**Micro (mg/kg DM)**					
Fe	142 ± 22.0	0.002	0.152	40.0 ± 0.00	40.0 ± 0.00
Cu	11.4 ± 1.6	0.001	0.012	9.00 ± 7.07	9.00 ± 7.07
Zn	50.0 ± 7.0	0.0009	0.055	14.5 ± 7.78	14.5 ± 7.78
Mn	100 ± 8.0	0.0008	0.095	22.5 ± 3.54	22.5 ± 3.54
Co	0.00003	0.00006	0.00003	0.115 ± 0.05	0.115 ± 0.05

*Sources: (16, 31–35)*.

## Phytochemical Composition of Hemp By-Products

Hemp has a total of 538 identified bioactive compounds dominated by terpenoids (>120), cannabinoids (>70) and polyphenols (11, 36, 43). Resin glands on trichomes or head cells of glandular hair are major production sites for terpenoids, cannabinoids, and polyphenols (26, 28). Terpenoids, cannabinoids, polyphenols, and fatty acids (FA) comprise classes of bioactive compounds of great interest in hempseed and its by-products due to their plethora of health-promoting properties (11, 43). Although cannabinoids including CBD and THC are not synthesized in seeds, they are transferred from resins, leaves and flowers into oil and oilseed cake during oil extraction (11, 27). Hence, cleaning and de-hulling of seeds are executed before oil extraction, to minimize cannabinoids transfer into oilseed by-products (11). Palade et al. (44) found traces of cannabinoids (i.e., 164.4 mg catechin equivalents (CE)/100 g of feed) in pig diets formulated with whole hempseed. Higher content of cannabinoids in hemp seed extracts is, therefore, a sign of contamination or use of medicinal cannabis varieties (27).

Other phytochemical constituents of hemp by-products include condensed tannins (CT), alkaloids, phenols, lignanamides, and tocopherols (35, 45). The CT are low in hempseed and HSC (Table 4). Hemp leaves might contain higher contents of CT since the concentration of bioactive compounds in hemp plant chronologically decreases from flowers, leaves, stems, seed to the roots (27). However, there is no available literature on CT of hemp leaves. Turner et al. (55) suggested that hempseed by-products contain alkaloids and this was confirmed by Yan et al. (56), but their contents have not yet been determined.

**Table 4:** Bioactive compounds and *in vitro* bioactivity profile of hemp by-products (Means ± SD).

	**Hemp by-product**			
	**Seed**	**Oil**	**Cake**	**Inflorescence**
**Phenolics (mg/kg DM)**				
Condensed tannins	1.10 ± 0.04	–	1.64 ± 1.87	
Catechin	–	498 ± 35.9	0.05 ± 0.03	51.1 ± 48.4
*N-trans*-caffeoyltyramine	490 ± 484	152 ± 11.2	–	38.2 ± 53.8
*p*-hydroxybenzoic acid	21.0 ± 12.7	78.6 ± 8.00	0.002 ± 0.001	–
Cannabisin A	1,051 ± 764	–	–	1.44 ± 2.02
Cannabisin B	–	64.9 ± 1.94	–	0.45 ± 0.07
Cannabisin C	–	–	–	0.19 ± 0.27
ferulic acid	–	47.4 ± 5.37	–	19.3 ± 23.1
Protocatechuic acid	10.0 ± 8.49	28.2 ± 2.47	–	–
TPC (mg GAE/g)	26.2 ± 36.0	1.23 ± 0.69	1.35 ± 1.87	31.5 ± 29.7
**Tocopherols (mg/100g)**				
γ-tocopherol	1,239 ± 1,076	516 ± 400	358 ± 28.9	–
α-tocopherol	44.1 ± 3.54	16.1 ± 5.33	29.7 ± 2.76	–
δ-tocopherol	281 ± 427	12.0 ± 4.00	11.3 ± 13.6	–
**Fatty acid (% Total FA)**				
Palmitic acid	6.19 ± 2.12	6.44 ± 1.99	7.54 ± 1.02	–
Stearic acid	2.61 ± 0.89	2.75 ± 0.84	3.21 ± 0.55	–
Oleic acid	11.6 ± 4.49	12.2 ± 4.49	12.7 ± 0.39	–
Linoleic acid	48.8 ± 17.6	50.2 ± 17.0	54.6 ± 1.56	–
γ-linolenic	2.61 ± 1.14	2.60 ± 1.16	2.97 ± 0.19	–
α-linolenic	14.9 ± 6.35	15.2 ± 6.47	17.2 ± 2.33	–
Total polyunsaturated fatty acid	66.7 ± 24.5	68.3 ± 22.7	75.4 ± 6.61	–
**Antioxidant capacity**				
DPPH (% inhibition)	45.8 ± 8.13	46.8 ± 0.00	31.1 ± 32.4	52.6 ± 35.4
ORAC (μmol TE/g)	127 ± 5.0	–	28.2 ± 6.19	–

*TPC, total phenolic content; DPPH, 1,1-diphenyl-2-picrylhydrazyl; ORAC, oxygen radical absorbance capacity; GAE, gallic acid equivalent; TE, trolox equivalent*.

*Sources: (3, 18, 35, 45–54)*.

Hempseed is a good source of lignan (320 mg/kg DM) (57, 58). It is dominated by lignanamides (cannabisin A) while HSC and inflorescences are dominated by flavanols (i.e., catechin; Table 4). More so, 99% of lignans are found in hempseed hulls, hence, dehulled hempseeds and the resultant cake have little lignan (57, 59). Syringaresinol content of hempseed hull (280 mg/kg DM) is the highest of any dietary source (57). Since HSC is produced from hulled seeds, its lignan content is expected to be low. However, there is limited literature on the lignan contents of HSC and leaves.

The tocopherol profile of hemp by-products is dominated by γ-tocopherol (Table 4), which is the tocopherol with the strongest antioxidant activity, but α-tocopherol is regarded as the most vital form (42, 60, 61). The α-tocopherol content of hemp by-products exceed dietary requirements for physiological function of growing small ruminants (10–20 mg/kg DM) and cattle (15–60 mg/kg) (33, 62), but below values (270–287 mg/kg) required to extend meat shelf life (63, 64).

Fatty acids in hempseed, oil, and cake contain 65–80% PUFA with the major FAs being linoleic (18:2 *n*-6) followed by α-linolenic acid (18:3 *n*-3) and oleic acid (C18:1 *n*-9; Table 4). This makes hempseed and its by-products an excellent source of essential fatty acids, with an omega 6 to omega 3 fatty acid ratio of ~3.3:1, which is similar to canola oil while providing a more healthful balance than soybean oil (7:1). Phytate (22.5 mg/g) and glucosinolates (3.8 μmol/g) are the most abundant anti-nutritional factors in HSC (18). However, at these low concentrations, phytate, and glucosinolates are unlikely to have adverse effects on ruminants (65, 66). Beneficial nutritional and phytochemical profiles of hemp by-products highlighted above provide a possible avenue for their inclusion in ruminant diets as protein sources, antioxidants and antimicrobials.

## *In vitro* Bioactivity of Phytochemicals in Hemp By-Products

### Antioxidant Activity

Hemp by-products contain potent antioxidants (Table 4), which decreases from flowers to leaves (27). The antioxidant capacity of hemp by-products, as measured by 1,1-diphenyl-2-picrylhydrazyl (DPPH) values (Table 4), are comparable to α-tocopherol (33.3–70%), a potent natural antioxidant commonly used commercially (67, 68). Hemp by-product oxygen radical absorbance capacity (ORAC) values are, however, lower than α-tocopherol (1,293 μm TE/g) (69), and ORAC values are thought to be more reflective of antioxidant capacity in biological systems (70).

Phenol amides (i.e., *N-trans*-caffeoyl-tyramine), lignanamides (i.e., cannabisin A, B, and C) (46, 71), tocopherols (42, 61) and CBD (11) are the major elements contributing to the antioxidant capacity of hempseed by-products. Chen et al. (71) and Irakli et al. (46) narrowed this list to *N-trans*-caffeoyl-tyramine, cannabisin A, B, and C as the major antioxidant phenolic compounds of hemp. Furthermore, Izzo et al. (45) confirmed that inflorescent extracts from hemp varieties with a high content of *N-trans*-caffeoyl-tyramine, cannabisin A, and B were more potent antioxidants. Overall, the current review highlights that hemp by-products are a rich and diverse source of potent antioxidants. However, there are still gaps in how this antioxidant potential may influence animal production, meat quality, shelf life, and sensory attributes. Further research is, therefore, required to ascertain their potency and mechanism of action during production, processing, storage/aging, display, and cooking through to consumption.

### Antimicrobial Activity

Essential oil extracts from the whole hemp plant material exhibit antimicrobial activity in most bacterial habitats from human, animal, and food sources, but are active against fungi (25, 72). Hempseed extracts have antimicrobial inhibitory effect on pathogenic bacterial strains of human origin (73). The highest hemp antibacterial activity is found in inflorescences (26, 74). Inflorescences are sites for the production of majority of bioactive compounds of hemp as they have resin glands (26, 28). With low THC and CBD contents in hempseed, antibacterial capacity of hempseed by-products could be attributed to terpenes, polyphenols and alkaloids. Terpinolene has been reported to be the main bioactive compound responsible for bacterial inhibitory activity of hemp inflorescence essential oil (74). Just like other monoterpenes, terpinolene disrupts bacterial cell membrane and wall integrity (75, 76). Generally, monoterpenes interact with bacterial cell membrane phospholipids, which results in increased permeability and leakage of cell content and ultimately cell death (75, 76). More scientific research into additive, synergic, and antagonistic antimicrobial effects of bioactive compounds in hemp by-products will be important to promote their use in the feed and meat industries.

Numerous studies have reported that polyphenols and alkaloids exert antibacterial properties through binding to the cell membrane hence inhibiting cell functions (77, 78). Overall, current literature shows that hemp whole plant essential oil extracts have good antimicrobial activity. However, to the authors' knowledge, no literature exists on the antimicrobial properties of various bioactive compounds found in hemp by-products and merits further investigation.

## Bioavailability of Bioactive Compounds of Hemp By-Products in Ruminants

Information on bioavailability of bioactive compounds is paramount in understanding their intake, digestion, absorption, metabolism, and excretion (80). That will, in turn, enable traceability of bioactive compounds and their derivatives in animal products (i.e., meat and milk). Bioavailability entails describing or quantifying the specific nutrient or bioactive available at a target organ/blood circulatory system level (81, 82). Bioavailability is an integral process that has five steps which include release from feed matrix in the gastrointestinal tract (GIT) (i.e., liberation), absorption, distribution, metabolism, and excretion.

To date, there is no literature on how digestion of hemp by-products in ruminants affects bioavailability of terpenes, cannabinoids, lignans, and polyphenols (Figure 1). Overall, terpenes and CBD are volatile compounds easily released from the feed matrix, absorbed and excreted unchanged (10, 83). Terpenes digestion and absorption begins during mastication and rumination and continues throughout the GIT (83). Glucuride conjugates of CBD were detected in urine and feces as the second most abundant component next to intact CBD in animal studies (10). These findings could imply that their metabolism during and/after uptake follows conjugation (phase II) processing with hydrophilic compounds such as glycine and glucuronic acid in the liver (28, 83). Less of phase II reaction of substances might occur in the gut or blood, but the majority occurs in the liver and bile due to abundance of enzymes involved in this reaction (28).

Metabolism pathways for terpenes and CBD are believed to be the same since the two compounds and their derivatives possess similar physical and chemical properties (27, 84). Previous studies demonstrated the transfer of dietary terpenes to ruminant meat (85–87). Since terpenes are fat-soluble, adipose tissue is the main depot assessed when studying between-diet effects in ruminants (87, 88). Cannabinoid conjugates were observed in major tissues of Large White pigs injected with THC (200 μg/kg) (84). Cannabinoids and their derivatives have also been detected in milk, feces, and urine of lactating ewes injected with THC as well as in fecal and urine samples of suckling lambs (89). Some studies concluded that cannabinoids are eliminated from the body after a short period (<48 h), even with chronic exposure (10, 84).

Kuhnle et al. (90) reported lignans in beef (6–16 μg/100 g wet weight) and lamb (4–17 μg/100 g wet weight). Ruminal biohydrogenation leads to the conversion of dietary lignans to enterolactones, which are absorbed from the gut and deposited in tissues including milk (91) and meat (90). Manipulation of dietary phenolic compounds has been confirmed to change their contents in meat (92, 93). However, not all phenolics found in the diet are incorporated in similar amounts in meat. Moñino et al. (92) observed that among the 11 major phenols identified in rosemary containing diets fed to lambs, only 3 (rosmarinic acid, carnosol, and carnosic acid) significantly increased with dietary inclusion levels of rosemary. Some of the phenolics in the diet are lost in feces or biotransformed before urinary excretion (92, 94).

The α-tocopherol content of meat from steers fed HSC diets was reported to be 2.55 mg/100 g lipid (95). Dietary α-tocopherols are non-degradable in the rumen (96), hence, availed in the small intestines for absorption and assimilation into adipose tissue and cell membranes to exert antioxidant activities (97, 98). Overall, bioefficacy of bioactive compounds is closely related to the amount released from the feed matrix, absorbed, and assimilated into tissues (80, 99). In this case, response/efficacy of bioactive compounds in meat derived from ruminants fed hemp by-products diets is closely related to their bioavailability. Understanding bioavailability of hemp by-products bioactive compounds is, therefore, essential in establishing their optimum inclusion levels in ruminant diets that efficiently improve meat production and quality.

## Effect of Hemp By-Products on Ruminant Nutrition

### Nutrient Intake

A survey by Bamikole and Ikhatua (100) indicates hemp leaves have been fed as an appetite stimulant in small ruminants. Feeding HSC either had neutral or positive effects on dry matter intake (DMI; Table 5). For example, feeding 1 kg or 1.4 kg/animal/day of HSC resulted in an increase in DMI for dairy calves, but not for steers (Table 5). The DMI increase for the dairy calves was attributed to reduced NDF rumen fill in HSC compared to SBM. Mustafa et al. (7) reported no differences in DMI of lambs fed diets containing 200 g HSC/kg DM, while Karlsson et al. (17) and Karlsson and Martinsson (79) included HSC in dairy cows (up to 320 g/kg DM) and lambs (218 g/kg DM) diets and recorded an increase in DMI. These inconsistences in DMI could be attributed to different inclusion levels, differences in composition of basal diets and animal species used across studies and deserve further investigation.

**Table 5:** Nutrient intake (Mean ± SD) of calves, steers and lambs fed hempseed cake, soybean, or canola meal diets.

	**Calves**			**Steers**			**Lambs**		
**Nutrient intake (kg DM)**	**HSC**	**SBM**	**SEM**	**HSC**	**SBM**	**SEM**	**HSC**	**CM**	**SEM**
DM	5.00	4.55	0.11	11.2	10.6	0.25	1.46	1.34	0.7
NDF	1.68	1.28	0.02	4.17	3.7	0.09	–	–	–
Starch	1.43	1.55	0.02	3.36	3.47	0.09	0.38	0.39	0.02
CP	0.83	0.64	0.01	1.43	1.24	0.02	0.14	0.12	0.007
Fat	0.16	0.09	0	0.26	0.18	0.4	–	–	–
ME^*^	58.6	53.7	1.25	134	127	3.1	11.3	11.6	0.45

* *MJ/kg DM; Calves, 6–8 weeks of age weighing 96 ± 21 kg; Steers, 13–15 months of age weighing 365 ± 28 kg; HSC, hempseed cake; SBM, soybean meal; CM, canola meal; DM, dry matter; NDF, neutral detergent fiber; CP, crude protein; ME, metabolisable energy; SEM, Standard error of mean*.

*Source: (6, 7, 79)*.

Inclusion of HSC (320 g/kg DM) in ruminant diets increased NDF and CP intake (6, 7, 17). It is of importance to note (79) reported that lambs fed HSC diet were able to attain required CP intake of 127 g/d to attain an average daily gain (ADG) of 250 g/d (32). It is not immediately clear how HSC and other hemp by-products influence nutrient intake when fed solely or in combination with other protein sources. This creates an opportunity for further studies on inclusion of hemp by-products in ruminant diets.

### Nutrient Digestibility

Hempseed cake has a low effective degradability of DM (EDDM) when compared to canola meal and SBM (Table 6). Dry matter digestibility is likely to be lesser for HSC vs. canola meal and SBM. However, HSC has been reported to increase rumen retention time and improve the rumen environment for microbial degradation as evidenced by fecal consistency scores (6). The CP solubility and degradability of HSC is low compared to canola meal and/or SBM (Table 6). However, potentially degradable protein portion of HSC is higher than canola meal and SBM. The aforementioned aspects result in increased ruminal passage of undegraded dietary protein (UDP). The UDP of HSC is highly digestible in the duodenum as compared to canola meal (7). Additionally, UDP and intestinal digestibility of HSC can be increased by moist heat treatment up to 130°C (109).

**Table 6:** Ruminal DM and CP degradation kinetics (Mean ± SD) of common oilseed by-products.

	**Hempseed cake**	**Soybean meal**	**Canola meal**
**Dry matter (DM)**			
Soluble (g/kg DM)	82.4 ± 3.36	307 ± 25.1	253 ± 34.6
Degradable (g/kg DM)	506 ± 6.21	684 ± 17.4	578 ± 21.9
Degradation rate (%/h)	2.40 ± 0.08	4.93 ± 1.91	5.13 ± 1.03
Effective degradability (g/kg)^a^	248 ± 2.81	665 ± 17.6	528 ± 49.6
**Crude protein (CP)**			
Soluble (g/kg CP)	65.3 ± 6.28	206 ± 48.6	195 ± 72.3
Degradable (g/kg CP)	901 ± 3.33	783 ± 50.6	715 ± 155
Degradation rate (%/h)	2.90 ± 0.17	4.77 ± 1.83	5.13 ± 0.81
Effective degradability (g/kg)^a^	394 ± 6.26	610 ± 30.6	525 ± 43.6
Rumen-undegraded CP (g/kg CP)	774 ± 9.47	415 ± 22.3	500 ± 36.1
Intestinally available CP (g/kg CP)	654 ± 11.9	–	342 ± 11.9
Total available CP (g/kg CP)	863 ± 8.45	–	869 ± 8.45
**Digestibility coefficient (g/kg)**			
Dry matter	640 ± 18.8	691 ± 6.35	683 ± 36.6
Organic matter	665 ± 18.3	707 ± 4.04	704 ± 28.2
Neutral detergent fiber	457 ± 21.2	460 ± 20.1	471 ± 28.7
Acid detergent fiber	330 ± 25.4	424 ± 28.3	352 ± 25.4
Crude protein	708 ± 8.61	689 ± 36.4	689 ± 0.71
**Ruminal fermentation parameters**	**Hempseed oil**	**Soybean oil**	**Canola oil**
pH	6.06 ± 0.12	6.36 ± 0.62	6.82 ± 0.39
NH3-N (mg/dL)	7.96 ± 0.84	11.8 ± 1.41	11.0 ± 1.46
Total VFA (m*M*)	37.4 ± 2.70	70.0 ± 62.3	78.7 ± 0.37
Acetate (m*M*)	14.8 ± 1.04	43.2 ± 33.4	42.3 ± 4.74
Propionate (m*M*)	10.9 ± 0.86	16.7 ± 17.6	17.0 ± 6.53
Butyrate (m*M*)	9.16 ± 0.56	9.51 ± 10.7	4.96 ± 3.28
Acetate: propionate	0.76 ± 0.10	3.45 ± 1.48	2.90 ± 1.01

a *Calculated at a rumen flow rate of 5%/h*.

*Sources: (7, 20, 101–108)*.

Digestibility coefficients of HSC are comparable to SBM and canola meal (Table 6). Hempseed cake has low NDF degradability (*in sacco*) because of its high indigestible NDF (iNDF; 409 g/kg DM) fraction (17, 110) which might be different *in vivo*. Although HSC has high EE (Table 2), which contributes to ME, fermentable energy to facilitate microbial growth is derived from fermentable carbohydrates (111).

Overall, hempseed oil has lower ruminal fermentation parameters compared to soybean and canola oil (Table 6). Total volatile fatty acids (VFA) can be as high as 200 mM just after feeding or decrease as low as 30 mM, however, its normal range is 70–120 mM (112). Low total VFA (37 mM) from hempseed oil could be an indicator of depressed rumen formation. Hempseed oil has a high PUFA content (Table 4), which may have an inhibitory effect on ruminal fibrolytic bacteria (113, 114). Hempseed oil reduces ruminal acetate production, which is generally an indicator of decreased fiber digestibility (101). Unaffected propionate production is an indicator of little or no influence on carbohydrate degradation (101). Hempseed cake has potential to maintain nutrient digestibility when used to replace other high-protein feedstuffs while hempseed oil depresses ruminal fermentation when added at levels <3.0 g/kg DM (101).

### Nitrogen and Methane Emissions

To the authors' knowledge, there are only two studies that evaluated *in vitro* ruminal nitrogen and methane production inhibition of hempseed by-products (101, 115). Findings of these studies showed that both whole hempseed and oil had neutral effects on ruminal ammonia-nitrogen production. Whole hempseed was 8% more effective at reducing methane than linseed but comparable to coconut oil (115). Embaby et al. (101) recorded a 10% decrease in methane production for hempseed oil compared to corn oil. Methane production reduction is attributed to high PUFA content in hempseed oil, which suppresses protozoa and acts as hydrogen sink through biohydrogenation (115, 116), with α-linolenic acid being a more potent anti-methanogen than linoleic acid (117). Whole hempseed is, however, more effective than oil at inhibiting methanogens since it has more terpenes, polyphenols and lignans, which are more toxic to methanogens than PUFA (118, 119). These compounds accumulate in cytoplasmic membranes as they are lipophilic thus disrupting methanogen cell membranes (118).

Although methane production is important in maintaining ruminal environment redox balance by providing a pathway for the excess pyruvate (112), it decreases the amount of ME obtained from a diet (69), hence, increasing energy required for meat production. The decrease in the concentration of methane produced may reduce atmospheric greenhouse gases and increase feed utilization efficiency as its emissions represent about 10% of gross energy loss from feed intake (120). Thus, reduction in methane emissions might maintain or improve animal performance by conserving energy which is redirected to animal growth (1). The limited available *in vivo* studies on antimethogenic effects of hemp by-products on animal performance warrants further research.

### Nutritional Disorders and Gut Health

Terpenes in hemp by-products have antibacterial properties (74). Specifically, terpinolene and oxygenated monoterpenes have been reported to strongly suppress rumen microbial activity in *in vitro* studies (121). Of importance, ruminal antimicrobial effects of terpenes *in vivo* might be lower than *in vitro* as terpenes are easily absorbed along the entire GIT reducing their concentration (83). Inhibitory effect of terpenes on undesirable microbial activity is useful in reducing the rate of ruminal fermentation and degradation to avoid nutritional disorders such as bloat, acidosis, and ruminal parakeratosis (120).

Levels of CT in hemp by-products are below recommended (20–50 g CT/kg DM) values reported to prevent bloat, acidosis, and parakeratosis (122, 123), except for leaves. CT bind to dietary protein forming complexes thereby reducing protein solubility, hence decreasing the chance of developing a stable rumen foam (123, 124). Additionally, CT have a bactericidal effect on bloat causing bacteria such as *Streptococcus bovis*, which produces a dextran slime that increases rumen fluid viscosity, hence, bloating (123, 124). CT exert bactericidal effects by binding to plant protein, forming a CT-bacteria cell wall interaction, inhibiting carbohydrate fermentation and proteolytic rumen bacteria (123). In turn, CT can also improve conditions for cellulolytic bacteria, and avoid acidosis and parakeratosis (123, 125).

Condensed tannins in hempseed by-products have the potential for suppressing gastrointestinal tract (GIT) nematodes (123, 124, 126) by inhibiting development of helminth eggs, reducing larvae, and adult motility as well as increasing the host animal's nutrient supply (123, 126). In that regard, hempseed by-products have the potential of improving gut health and preventing nutritional disorders among ruminants.

## Growth Performance, Carcass and Physicochemical Meat Quality Attributes of Ruminants Fed Hemp By-Products

Karlsson and Martinsson (79) observed low growth performance of lambs fed HSC compared to canola meal. For this study, lambs on the HSC diet had a high CP intake with a low ME intake (Table 7). A ME and CP intake balance are required for the animal to gain weight (33). Excess CP intake creates an excretion burden, thereby affecting ADG and final live weight (32, 33). However, feeding whole hempseed to steers (19) or HSC to growing cattle (6), and dairy cows (17) did not affect final live weight or ADG. Lack of differences in animal growth when feeding HSC or SBM/canola meal to ruminants could be attributed to their similarity in chemical composition, nutrient intake and digestibility (Tables 2, 3).

**Table 7:** Growth performance of lambs and steers fed hempseed cake or other protein feed.

	**Lambs**			**Steers**		
**Attributes**	**HSC**	**CM**	**SEM**	**HS**	**SBM**	**SEM**
Total gain (kg)	6.4	9.5	0.52	192.6	193.3	0.95
Average daily gain (kg/d)	119	175	9.6	1.16	1.16	0.00
Body condition score (1-5)	2.9	3	0.04	–	–	
Feed conversion (DM/gain)	7.9	5	0.37	0.133	0.133	0.00

*HSC, hempseed cake; CM, canola meal; HS, hemp seed (full-fat); SBM, soybean meal; SEM, Standard error of mean. Inclusion levels: HSC−218 g/kg DM, CM−254 g/kg DM, and HS−140 g/kg DM*.

*Source: (19, 79)*.

Overall, feeding whole hempseed (14% as fed) and HSC (1.4 kg/animal/day) had neutral effects on carcass and meat quality traits in feedlot steers (19, 95) and lambs (127). These findings are consistent with nutrient intake, digestibility, and animal growth data reported for the hempseed by-products in the current review. Similarly, feeding HSC resulted in comparable FA profiles with SBM and canola meal for beef and lamb meat, which was dominated by MUFA (oleic acid; C18:1) (Table 8) and these similarities are related to their dietary FA composition. Overall, HSC has comparable effects compared to SBM on ruminant growth performance, carcass, and physicochemical meat quality attributes. However, no information is available on volatile compounds or sensory attributes of meat from ruminants fed hemp by-products, and this requires investigation.

**Table 8:** Fatty acid profile (Mean ± SD) of *longissimus dorsi* from steers and lambs fed hempseed cake, canola, or soybean meal.

	**Steers**		**Lambs**	
**Fatty acids (% Total FA)**	**HSC**	**SBM**	**HSC**	**CM**
Total fat content (g/100g)	10.6 ± 4.18	7.50 ± 2.14	3.70 ± 0.67	3.6 ± 0.67
Palmitic acid	28.3 ± 1.17	31.0 ± 2.65	23.0 ± 1.48	22.1 ± 1.48
Stearic acid	13.1 ± 0.71	12.8 ± 0.83	14.7 ± 1.24	13.8 ± 1.24
Oleic acid	44.9 ± 1.16	41.8 ± 2.78	37.4 ± 2.06	41.2 ± 2.06
Linoleic acid	1.20 ± 0.10	1.36 ± 0.48	4.49 ± 0.55	5.84 ± 0.55
γ-linolenic	–	–	0.04 ± 0.01	0.06 ± 0.01
α-linolenic	0.25 ± 0.03	0.21 ± 0.05	0.61 ± 0.09	0.78 ± 0.09
Total saturated fatty acids	44.6 ± 1.09	47.4 ± 3.46	41.3 ± 2.80	42.4 ± 2.80
Total monounsaturated fatty acids	51.4 ± 1.26	48.4 ± 2.75	48.2 ± 2.29	44.9 ± 2.29
Total *n*-6 fatty acids	1.52 ± 0.49	1.80 ± 0.70	5.79 ± 0.72	7.48 ± 0.72
Total *n*-3 fatty acids	0.37 ± 0.11	0.36 ± 0.15	1.26 ± 0.15	1.54 ± 0.15
Total polyunsaturated fatty acids	2.08 ± 0.56	2.28 ± 0.85	8.33 ± 0.85	10.59 ± 0.85

*HSC, hempseed cake; SBM, soybean meal; CM, canola meal*.

*Source: (95, 127)*.

## Aging-Proteome Changes and Shelf Life of Meat From Ruminants Fed Hemp By-Products

Proteomics is a relatively new technique in meat science that provides an avenue for understanding meat tenderness and color stability with respect to proteins involved at a molecular basis (128, 129). The technique does not only enable identification of myofibrillar proteins, protein, and glycolyticc enzymes involved in meat tenderness and color stability, but also allows establishment of the relationship between these proteins and bioefficacy of meat bioactive compounds (128, 129). Nassu et al. (130), for example, found that high muscle α-tocopherol content protects meat discoloration at longer aging days (21 d), but does not affect meat tenderness. However, no proteomics was done in this study. The relationships between major meat bioactive compounds in hemp by-products and meat aging have not been investigated. Understanding how these antioxidant bioactive compounds interact with muscle protease system is crucial in establishing their impact on meat quality during aging.

*In vitro* studies show that hemp has antimicrobial properties (25, 74) due to its moderate contents of terpenes, CBD, α-tocopherol, and polyphenols, which could be transferred into ruminant meat (92, 93). Hempseed increases meat PUFA content (19), which could make it susceptible to lipid oxidation. To authors' knowledge, no studies have evaluated the impact of hemp by-products in ruminant diets on myoglobin, lipid, and protein oxidation. However, feeding HSC increased total antioxidant capacity of sheep milk (47) owing to the moderate to high terpenes, CBD, α-tocopherol and polyphenol contents in the diet, which are transferable to tissues. Thus, feeding hemp by-products could have positive effects on meat oxidative stability, and merits research.

## Further Research

Increasing consumer demand for hemp products is driving a wave of regulatory changes allowing its commercial production globally. Consumers generally perceive hemp products such as fiber, seed, seed oil, CBD oil, and CBD fortified commodities as organic and healthy, hence, are willing to pay a premium for them (4). Hemp leaves, seed, hulls and HSC have potential as livestock feed and meat preservatives. *In vitro* studies suggest antimicrobial and antioxidant properties of hemp bioactive compounds, which are yet to be affirmed *in vivo*. Some studies have already included hemp by-products as protein sources in finishing diets for goats, sheep, cattle, and monogastrics (3, 6, 95, 127). Either neutral or superior animal health and performance attributes for hemp by-product fed animals compared to conventional oilseed cakes were reported (6). However, there is still a gap in understanding the impact of hemp by-products on nutrient digestibility, nitrogen, and methane emissions, nutritional disorders, gut health, and meat quality, thus more research is warranted.

Overall, nutrient and bioactive compounds in hemp by-products are biologically accessible in the GIT and available in the animal body system (109), but details on their bioavailability are incomplete. These bioactive compounds have been identified in the circulatory system, muscle, and brain tissue, feces and urine in animal models (84). Retention of these bioactive compounds in milk has been investigated (89), but for meat, it is yet to be determined. If these bioactive compounds are retained in meat, it would be important to determine their efficacy in enhancing keeping and eating qualities of meat. Bioavailability of bioactive compounds of hemp by-products could be determined using *in vitro* digestion, *in-vivo* and/or *ex-vivo* using blood and organs, respectively (80, 99). Various techniques have been developed to assess ruminal degradability and intestinal digestibility of feed ingredients *in-vitro* (131, 132). Distribution of hemp by-products' bioactive compounds among major tissues can also be determined *ex-vivo* using the GC-MS procedure (133). The overall challenge in ruminant production is estimating the transfer of bioactive compounds from hemp by-products into meat and establishing their bioefficacy in improving animal health and production as well as keeping and eating qualities of meat.

## Author Contributions

FS: drafted the manuscript with editorial inputs from CK, OC, MM, and CM. CM: conceptualized the review and acquired funding. All authors contributed to the article and approved the submitted version.

## Conflict of Interest

The authors declare that the research was conducted in the absence of any commercial or financial relationships that could be construed as a potential conflict of interest.
